# Does the Stimulus Type Influence Horses’ Performance in a Quantity Discrimination Task?

**DOI:** 10.3389/fpsyg.2012.00504

**Published:** 2012-11-16

**Authors:** Yuki Henselek, Julia Fischer, Christian Schloegl

**Affiliations:** ^1^Cognitive Ethology Laboratory, German Primate CenterGöttingen, Germany

**Keywords:** quantity discrimination, stimulus type, horses, edible reward, inedible reward

## Abstract

The ability to understand the relation between quantities has been documented in a wide range of species. Such quantity discrimination competences are commonly demonstrated by a choice of the larger quantity or numerosity in a two-choice task. However, despite their overall success, many subjects commit a surprisingly large number of errors even in simple discriminations such as 1 vs. 3. Recently, it had been suggested that this is a result of the testing procedure. When monkeys could choose between different quantities of edible rewards, they showed low-level success. If, however, they chose between inedible items and were rewarded with edible items, their performance increased. The same held true if they chose between edible items but were rewarded with other edible items (Schmitt and Fischer, 2011). This led to the suggestion that the monkeys may not have been able to mentally separate between choice- and reward-stimuli in the initial test situation. To investigate if this response pattern can also be found in non-primate species, we replicated the experiment with 12 Icelandic horses kept at a private horse-riding school. Horses are known to discriminate between quantities up to three, but are very distantly related to primates. Unexpectedly, we found only weak evidence for quantity discrimination skills and no effect of the type of stimuli. Only some subjects reliably selected the larger quantity in some, but not all quantity pairs. These findings are not only in contrast to the previously conducted study on monkeys, but also to other studies on horses. From this, we conclude that quantity discrimination competence may only be of minor importance for horses and highlight the influence of experimental conditions on the outcome of cognitive tests.

## Introduction

The ability to understand the relation (i.e., greater or smaller) and to discriminate between two or more quantities is of particular interest in comparative cognition (see, for instance Agrillo and Petrazzini, 2012). Quantity discrimination competences can be used to differentiate between different amounts of items and may be based on differences in volume, surface, etc. (e.g., Agrillo et al., 2010b). If the discrimination is based on the numerosity of the items rather than any on the alternative measurements, researchers often refer to this as numerical competence (e.g., Pepperberg and Gordon, 2005; Beran, 2008; Agrillo et al., 2009). In either case, such discriminatory skills can be used in different ecological contexts such as foraging or in conflicts. For instance, New Zealand robins, *Petroica australis*, used their mental representation of numerosities to maximize the intake of retrieved cached food (Hunt et al., 2008) and female lions, *Panthera leo*, approached a group of intruders only if their own group was larger than the intruder group (McComb et al., 1994). In general, quantity discriminations and numerical competence have been described in a large number of animals, ranging from chicks *Gallus gallus* (Rugani et al., 2009), mosquitofish *Gambusia holbrooki* (Agrillo et al., 2009), horses *Equus caballus* (Uller and Lewis, 2009), and black bears *Ursus americanus* (Vonk and Beran, 2012) to capuchin *Cebus apella* (Evans et al., 2009) and rhesus monkeys *Macaca mulatta* (Flombaum et al., 2005; Brannon et al., 2006), chimpanzees *Pan troglodytes* (Boyson and Berntson, 1995) as well as corvids *Corvus corax*, *C. corone cornix*, and *C. macrorhynchos* (Koehler, 1943; Smirnova et al., 2000; Bogale et al., 2011), gray parrots *Psittacus erithacus* (Pepperberg, 1987; Pepperberg and Gordon, 2005; Al Aïn et al., 2009), and pigeons *Columba livia* (Scarf et al., 2011).

The vast majority of quantity discrimination tests rely on some version of two-choice task, in which the subjects are confronted with a choice between different quantities of rewards, of which they commonly shall choose the larger quantity. In a subset of these studies, arbitrary objects are used as stimuli (e.g., Pepperberg and Gordon, 2005; Beran, 2008; Agrillo et al., 2009, 2010a; Vonk and Beran, 2012), often in conjunction with presentations on touch screens. These procedures facilitate testing for true numerical competence as it easily allows to control for confounding variables such as the volume or surface of the stimuli (Agrillo et al., 2010b). Other procedures use pieces of food as a reward (e.g., Al Aïn et al., 2009; Uller and Lewis, 2009; Schmitt and Fischer, 2011) or, in a few cases, different numbers of conspecifics (e.g., Dadda et al., 2009) or nestmate surrogates (i.e., objects chicks had been imprinted on; Rugani et al., 2009). These procedures have the advantage of greater ecological relevance and require less training.

There is an interesting discrepancy in tests involving food rewards as choice stimuli. Some studies report high discrimination performances (e.g., Evans et al., 2009 for capuchin monkeys, Beran and Beran, 2004 for chimpanzees, or Al Aïn et al., 2009 for Gray parrots), whereas in other studies subjects performed surprisingly poorly. For instance, Western lowland gorillas *Gorilla gorilla gorilla* required training to succeed (Anderson et al., 2005); chimpanzees, orangutans *Pongo pygmaeus*, olive baboons *Papio anubis*, and long-tailed macaques *Macaca fascicularis* succeeded in 65–70% of the trials only, even in relatively simple discriminations such as 1 vs. 2 (Herrmann et al., 2007; Schmitt and Fischer, 2011). Schmitt and Fischer (2011) suggested that this may be a consequence of the usage of food as choice stimulus *and* reward and highlighted two confounding and not necessarily mutually exclusive problems: impulse control and mental representation of choice and reward. The first issue, impulse control, arises if the perception of food (as choice stimulus) leads to an impulsive choice of any reward without evaluation of the two alternative options. Problems in self-control have been reported repeatedly in non-human animals (e.g., Stevens et al., 2005; reviewed by Fawcett et al., 2012). The second issue, the mental representation of choice and reward, has been highlighted in human psychology. Humans readily form “dual representations,” i.e., upon seeing a picture of a famous person the picture is represented simultaneously as a picture and as the person that is depicted. When young children are confronted with stimuli of high physical salience (e.g., candies), it obstructs their ability to see them as choice item rather than as reward (DeLoache, 2000). To illustrate this: when 3-year-old children were tested in a reversed-reward paradigm, they performed significantly better if they had to choose between symbols (i.e., a mouse or an elephant) than when they had to choose between different types of candies (Carlson et al., 2005); this has been interpreted as children’s inability to identify the two different functions of the highly salient candies, i.e., choice stimuli vs. reward. Thus, it seems plausible that also non-human animals may have problems to mentally represent the food once as choice stimulus and once as the reward (DeLoache, 2000; Shifferman, 2009).

To evaluate their hypothesis, Schmitt and Fischer (2011) used a standard two-choice paradigm to test the quantity discrimination abilities of olive baboons and long-tailed macaques. The authors applied three conditions. In the first condition, raisins served as choice and as reward. In the second condition, the monkeys were presented with pebbles and were rewarded with the quantity of raisins corresponding to the number of pebbles they chose. Thus, the choice stimuli were inedible items but they were rewarded with edible items. In the third condition edible items were used as choice and as reward, but the subjects received not the raisins they chose, but the same amount of other raisins. The monkeys selected the larger quantity in all three conditions, but they performed significantly better when they were not rewarded with the items they had to choose. Importantly, their performance was identical in the condition in which they chose between pebbles and in the condition in which they chose between raisins, but were rewarded with other raisins. This suggests that the relatively poor performance in the first condition (in which they were rewarded with the same raisins they had chosen) cannot be explained solely by problems with impulse control; it rather suggests that the monkeys indeed had problems to mentally represent the raisins as two different things at the same time, namely as choice stimulus and as reward (Schmitt and Fischer, 2011).

We here replicated the original Schmitt and Fischer (2011) – study with horses. The cognitive abilities of horses received increasing attention recently: they can recognize familiar horses and humans cross-modally (Proops et al., 2009b; Lampe and Andre, 2012; Proops and McComb, 2012), identify the attentional states of humans (Proops and McComb, 2010; Krüger et al., 2011), and respond at least to some human-given social cues (McKinley and Sambrook, 2000; Proops et al., 2010; Krüger et al., 2011). Of more relevance for our purpose are studies demonstrating that horses can discriminate objects of different colors (Smith and Goldman, 1999) and sizes (Hanggi, 2003). The first claims of numerical or even mathematical skills in horses have been refuted and became the text-book example of behavioral cueing and the need for adequate controls for experimenter biases (“Clever Hans”; Pfungst, 1911); nevertheless, a recent study demonstrated that horses can indeed discriminate between different quantities (Uller and Lewis, 2009). In this study, an experimenter put several apples, one by one, in two buckets. Then, holding the buckets in her hand, the experimenter approached the horses to allow them to make a choice. The results demonstrate that the horses managed a 1 vs. 2 and a 2 vs. 3, but not a 4 vs. 6 discrimination.

Interestingly, horses’ cognitive performances seem to be highly susceptible to minor changes in testing conditions (see Proops et al., 2009a and references therein), making them an interesting subject for comparisons of experimental approaches. Thus, in the present study, our intentions were twofold. First, we were interested whether the same effect that had been described in the monkeys (Schmitt and Fischer, 2011) would also be found in a non-primate species. Second, we wished to expand on the previous study by Uller and Lewis (2009) on quantity discriminations in horses; thereby we purposefully did not replicate that study in detail, but instead applied a different methodology closely resembling the monkey-study by Schmitt and Fischer. By doing so, we hoped not only to investigate if horses could also discriminate quantities beyond those tested by Uller and Lewis, but also to investigate the robustness of horses’ performances when a different methodological approach is used (Proops et al., 2009a). The value of such approaches for comparative animal cognition research is increasingly acknowledged (e.g., Agrillo and Petrazzini, 2012; MacLean et al., 2012) as some authors had argued that much can be learned from subjects’ failures in cognitive tests (e.g., Thornton and Lukas, 2012). Importantly, the goal of this approach is not to discredit or devalue earlier studies, but to identify yet unknown factors contributing to the subjects’ performances, which ultimately can support the development of better testing regimes (e.g., Mulcahy and Hedge, 2012; Seed et al., 2012). For instance, after the original finding that chimpanzees would demonstrate perspective taking skills (Hare et al., 2001), follow-up studies revealed that this was restricted to certain test conditions and depended, among others, on the size of the testing compartments (Karin-D’Arcy and Povinelli, 2002; Bräuer et al., 2007); this highlighted the importance of an so far vastly neglected factor in the design of cognitive tasks. More recently, varying evidence for elephants’ numerical competencies was found, again depending on the precise testing conditions (Irie and Hasegawa, 2012; Perdue et al., 2012).

For our study, the horses were confronted on each trial with a choice between two different quantities, and we applied the three conditions used by Schmitt and Fischer (2011; see above). A total of six different quantity pairs of varying ratio (i.e., larger quantity divided by smaller quantity) and quantity difference (larger quantity minus smaller quantity) were presented to evaluate the occurrence of two frequently observed patterns in quantity discrimination tasks: first, the discriminability increases with an increase in ratio (e.g., 2 vs. 4 is easier to solve than 2 vs. 3; Weber’s law) and second, when keeping the ratio constant, discriminations become harder when the total quantities increase (i.e., 4 vs. 6 is harder than 2 vs. 3). Thus, particularly when ratios are small, differences between large quantities become difficult to assess.

Based on the published record, we expected horses to be able to discriminate between different quantities and that their performance would obey Weber’s law. Furthermore, we predicted that if the horses’ performance would be influenced by the stimulus type, their performance should be better when choosing between inedible rather than edible items. If this would be based on a facilitated impulse control, an increased performance should be detectable with inedible items as choice stimuli only. If, however, the horses’ mental representation of the stimuli would benefit from the separation of food as choice stimuli and food as reward, an increased performance could be expected in the condition in which they would choose between different food quantities but would be rewarded with other rewards.

## Materials and Methods

### Subjects

We tested 12 privately owned Icelandic horses (six gelded males, six mares) kept at a private horse-riding school near Göttingen, Germany. At the time of testing, the subjects were between 6 and 18 years old. The horses were housed individually in open stables and were frequently ridden. Tests were conducted between December 2011 and February 2012. As the horses were used for horse-riding courses, the frequency of testing depended on the availability of open time slots. Horses were fed daily and hay was available *ad libitum*. Additional feeding with grains usually occurred after riding, but tests were conducted before riding lessons. However, some of the horses were privately owned and, unbeknown to us, the owners may have grain-fed their horses on some days prior to testing. Tests were conducted once to thrice a week. Each horse participated not more than once per day; sessions were conducted between 10 a.m. and 4 p.m.

### General procedure

**Figure 1:**
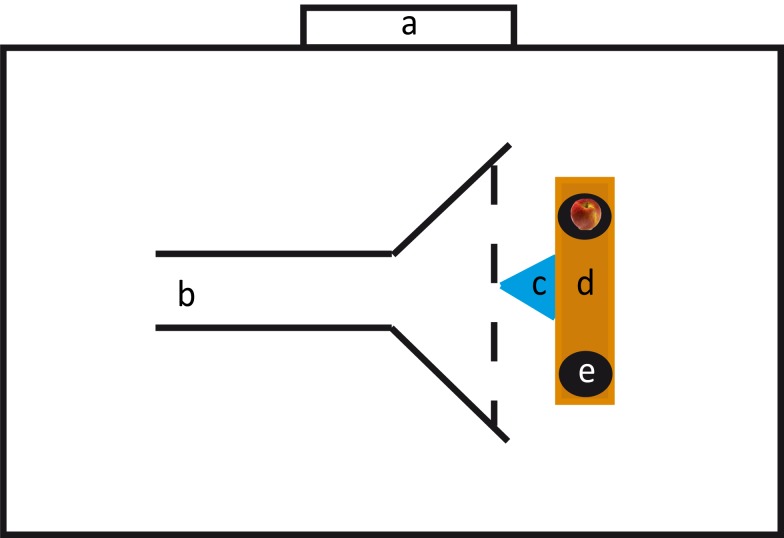
**Experimental setup**. a: Exit, b: corridor, c: multi-jump block, d: bench, e: bucket.

Testing was conducted in the center of a large indoor riding arena. We built a corridor through which the horses would approach the setup. This corridor was 7.4 m long, 1.70 m wide in the beginning and opened after 3.30 m to a width of 3.90 m (Figure 1). To the sides the corridor was confined by barrier tape, but was open to the back and to the front. At the end of the corridor we positioned a 2.20 m long bench; at both ends of the bench we put a flat bucket upside down (diameter: 30 cm) on which the stimuli were presented. The distance between the two buckets was 1.35 m. In front of the bench and equidistantly between the two buckets we positioned a multi-jump block (1 m long and 0.60 m high) made of plastic. This block served as hindrance for the horses to approach the experimenter and induced them to approach one of the buckets.

Each trial involved two experimenters. Experimenter 1 (Yuki Henselek) sat behind the bench, baited the buckets, and rewarded the horses. Experimenter 2 led the horse. Three different persons served as experimenter 2 (two females, one male, all of comparable age). All horses were tested with all experimenters. At the beginning of a trial, experimenter 2 led the horse from the back of the arena into the corridor to the point where the corridor widened. This was defined as the release point. Experimenter 2 led the horse on its left side; at the release point, the experimenter let the horse go and made a few steps back to stand behind the horse. Five horses had a significant side bias (according to a binomial test with *P* < 0.05) and three horses chose preferentially the bucket to their left and two horses the bucket to their right. Thus, it seems unlikely that position of experimenter 2 biased the horses to select one side consistently. The horse approached the bench to make a choice. After the horse had chosen, experimenter 2 approached and led it outside of the corridor to the backside of the arena. There, the horse was led on a pre-defined, eight-shaped course through the arena to counteract potential side-biases (Proops et al., 2010). During this, experimenter 1 prepared the next trial, which started when experimenter 2 and the horse re-entered the corridor.

### Pre-test

The pre-test was introduced to ensure that the subjects were familiar with having to make a choice. On each trial, experimenter 1 put one apple slice (approx. 1/8 of an apple) on one of the buckets, whereas the other bucket remained empty. Because of the high olfactory acuity of horses (Saslow, 2002) we decided to control for olfactory cues by hiding four slices of apple under each bucket. The location of the single apple slice (right or left) was randomized with the stipulation that it was not on the same side in more than in two consecutive trials. Although the horse was distracted by going the eight-shaped course it could not be excluded that the subject may have seen the experimenter’s movement toward the bucket during baiting. Therefore, experimenter 1 held her arms extended from her body and moved both arms simultaneously to both buckets. Nevertheless, because of the distance between the buckets, the experimenter touched the buckets sequentially; we randomized whether the experimenter touched the baited bucket first or second. After the baiting, experimenter 1 held her arms and hands attached to her body, lowered her head, and turned around by 180° to avoid giving unintentional cues. The horse was scored as having made a choice if it overstepped the imaginary horizontal line of the block with at least one hoof (see Figure 1).

One session consisted of 10 trials. When subjects were successful in nine out of ten trials in a single session or were correct on 80% of their last 20 trials (binomial test, two-tailed, in both cases *P* < 0.05), they were advanced to the test.

### Test

The general test procedure was identical to the pre-test with the following exceptions: at the beginning of each session the horse was led through the corridor and to each of the two buckets once. After this warm-up, the test began. Here, the subjects had to choose between different quantities of rewards. All rewards were of comparable size and consequently, number was positively correlated with several other parameters (e.g., volume and surface). We presented six different quantity pairs representing three different ratios. The quantity differences ranged from 1 to 4 (see Table 1).

**Table 1:** **Quantity pairs used in the experiment, grouped by the ratios**.

Ratio	1:2	1:3	2:3
Quantity pairs	1:2 (1)	1:3 (2)	2:3 (1)
	2:4 (2)	2:6 (4)	4:6 (2)

*Values in parenthesis represent the value differences*.

Each session consisted of twelve trials and each quantity pair was presented twice per session in randomized order. Furthermore, we conducted three different conditions:

Condition “food”: the apple slices served as choice stimuli and as reward; i.e., the horses retrieved the apple pieces from the bucket.
Condition “wood”: small wooden blocks (same size as the apple slices) served as choice stimuli. As soon as a horse had made a choice, the experimenter removed the wooden blocks and provided the horse with an amount of apple slices corresponding to the number of wooden block the horse had chosen. The reasoning behind this task was that if the horses had understood to approach the larger quantity, they should continue to do so even if non-food items served as stimuli (and thus behave like the monkeys in the original study).
Condition “food replaced”: apple slices served as choice stimuli, but subjects were rewarded with other pieces of apple. Thus, as soon as a horse had made a choice, the experimenter removed the apple slices and provided the horse with an amount of apple slices corresponding to the number of apple pieces the horse had chosen.

During the first session, we encountered that we had to modify the setup in two ways. First, to facilitate the positioning of the choice stimuli and the replacement of the choice stimuli with the rewards, the choice stimuli were positioned on trays (same diameter and color as the buckets). Thus, the experimenter prepared the stimuli on the trays and positioned the trays with a single movement on the two buckets. As soon as the horse made its choice, the entire tray was removed and the horse rewarded. Second, it turned out that the experimenter needed to be orientated toward the horse (rather than turning her back toward them) to allow the experimenter to remove the trays in time. To avoid cuing, the experimenter was looking down until the horse had made its choice. Two horses (one in condition “food” and one in condition “food replaced”) were first tested in the procedure described for the pre-test and were tested with this new procedure from the beginning of their second session. However, as their performance did not differ between the two treatments, we did not repeat the first session for these two horses.

Conditions were presented en bloc and we conducted four sessions for each condition. Because of time constraints, each horse was tested in two of the three conditions only. We formed three groups (group 1 tested in conditions “food” and “wood,” group 2 in conditions “food” and “food replaced,” and group 3 in conditions “wood” and “food replaced”) and randomly assigned four horses to each of the groups. Within each group, half of the horses began with one condition and the other horses began with the other condition. Thus, each horse received eight sessions with a total of 96 trials (12 trials per session).

### Statistical analysis

Sessions were video-recorded and analyzed from tape. Due to technical problems, the performance on 3 days had to be scored live. Twenty percentage of all sessions were re-coded by a second person and the inter-observer reliability was excellent (Cohen’s *K* = 1).

We identified a variety of task-related factors that may have influenced the performance of the horses, ranging from the different conditions to the inevitable use of several persons assisting in the role of experimenter 2. We used a general linear mixed model (GLMM) to test the influence of these factors. This approach has several advantages: first, the influence of each factor can be assessed while keeping the other factors constant. Second, this approach is robust to missing values (note that each horse participated in two of the three conditions only); third, it reduces the number of statistical tests and thereby the risk of a type I error. We used choice of the larger quantity as the binomially distributed dependent variable. Condition, quantity pair, trial number within each session, identity of experimenter 2, location of the higher quality (left/right), and the order of touching the buckets (positioning the larger or the smaller quantity first) were entered as fixed factors; subject ID was entered as random factor. In concordance with standard stepwise model reduction procedure, we sequentially removed all fixed terms in order of decreasing significance, whereby the least significant term was determined after each removal step (Galwey, 2006; Garamszegi et al., 2009). Deletion of fixed terms continued until only terms with a significance value below 0.1 remained. This was then considered the final model. Excluded terms were re-entered one by one into the final model to confirm that they did not explain a significant part of the variation.

Individual performances were assessed using binomial tests. To compare the group performance against the chance level, we used one-sample *t*-tests or Wilcoxon-tests, depending on the distribution of the data (according to a Shapiro–Wilk – test for normality). To test if the horses’ performances differed between different ratios or quantity differences, we used repeated measures ANOVAs; we applied the Greenhouse–Geisser correction in case of a lack of sphericity (according to Mauchly’s test). Sex differences were analyzed using a *t*-test. Finally, Pearson correlations were calculated to assess performance changes over the course of the experiment, age effects, and a relationship between test and pre-test performance. All tests were conducted two-tailed with alpha set to 0.05. Tests were conducted using SPSS19.

## Results

Nine of 12 subjects reached the criterion in the Pre-Test and were advanced to the test. Successful subjects required 3.77 ± 2.49 (*x* ± SD) sessions to reach the criterion. One horse in each group stopped participating and refused to make a choice.

**Figure 2:**
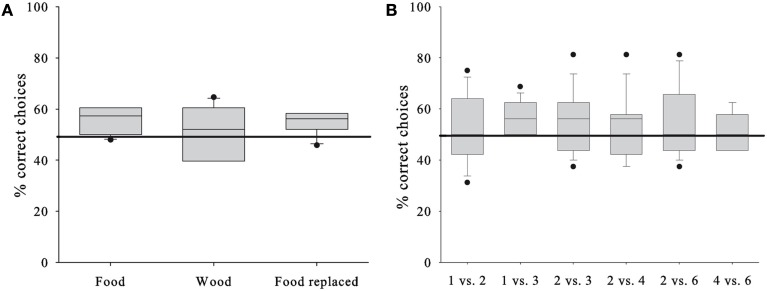
**Horses’ test performances**. **(A)** Percentage of correct choices in each of the conditions. **(B)** Percentage of correct choices in each of the six quantity pair discriminations. Box plots show median and 25th and 75th, percentiles, whiskers show 10th- and 90th percentiles and dots represent outliers. Horizontal line represents chance level.

The GLMM did not reveal a significant contribution to the subjects’ performance by any of the factors (see Table 2 for the full model). This suggests that in contrast to our expectations the horses’ performance was independent from the stimulus presentation (*P* = 0.606), i.e., whether the horses chose between the rewards they were going to receive, inedible items, or edible items that were going to be replaced (Figure 2A). Consequently, we pooled the conditions for subsequent analysis. Even more surprisingly, the performance did not differ between the various quantity pairs (*P* = 0.893); nevertheless, we found an above chance performance for the presumably easiest 1 vs. 3 – pair (one-sample *t*-test, *T* = 3.162, df = 8, *P* = 0.013), but one needs to bear in mind that the median success rate was below 60% (Figure 2B) and the observed effect could be a chance finding. The against chance comparisons for all other quantity pairs turned out to be non-significant (one-sample *t*-tests, *P* > 0.249; Wilcoxon-Test, *P* = 0.672; Figure 2B).

**Table 2:** **GLMM test statistics of the full model**.

Factor	df	*F*	*P*
Condition	2	0.501	0.606
Quantity pair	5	0.333	0.893
Trial number within session	11	0.193	0.661
Identity of experimenter 2	1	0.105	0.745
Location of higher quantity	1	1.711	0.191
Order of touching the buckets	1	0.193	0.660

Our results furthermore fail to demonstrate an effect of ratio (rm ANOVA, *N* = 9, *P* = 0.718, *F*_2,16_ = 0.339) or quantity difference (rm ANOVA, *N* = 9, *P* = 0.871, *F*_1.255,10.038_ = 0.055) on the subjects’ choice behavior. On an individual level, one subject each selected the larger quantity in the 2 vs. 3 – pair, the 2 vs. 4 – pair, and the 2 vs. 6 – pair (in all cases 13 out of 16 trials correct; binomial test: *P* = 0.021). Two of these three horses also had a marginal preference for the larger quantity (12 out of 16 correct, *P* = 0.077) in the 2 vs. 6 and the 1 vs. 2 – pair, respectively.

**Figure 3:**
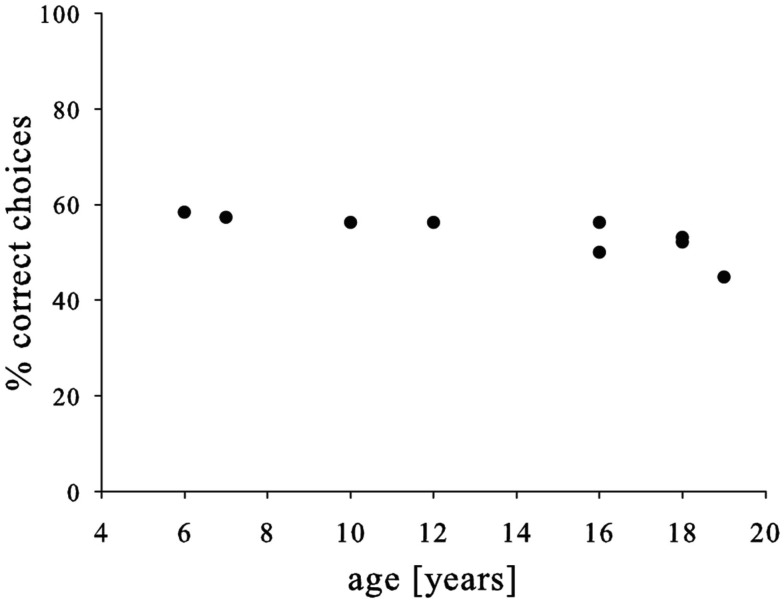
**Relationship between age of the subjects and choice accuracy**.

Finally, we neither found a link between pre-test and test-performance (Pearson, *N* = 9, *P* = 0.202, *R* = 0.469) nor an improvement over the course of the experiment (Pearson correlation, *N* = 8, *P* = 0.959, *R* = −0.022). Similarly, males and females performed similarly (*t*-test, *N* = 9, *P* = 0.696, *T* = −0.408), but we found a somewhat weaker performance in older subjects (Pearson, *N* = 9, *P* = 0.014, *R* = −0.774; Figure 3). Yet, again the effect was not very strong and mainly driven by the oldest subject; consequently, the strength of this conclusion should be treated with caution.

## Discussion

In contrast to our expectations and to a previous report (Uller and Lewis, 2009) we did not find evidence that horses will discriminate between two different quantities. Only a few subjects selected the larger quantity consistently, but only in a subset of the quantity pairs. Unfortunately, no clear pattern emerged, as some of these subjects solved presumably more difficult discriminations (e.g., 2 vs. 4) but failed in presumably more simple discriminations (e.g., 1 vs. 3). As quantity pairs were presented in randomized order varying degrees of motivation appear unlikely to explain these findings.

Interestingly, the horses were indifferent to the type of choice stimuli that were presented, i.e., they were not more or less successful when they chose between slices of apple than when they chose between wooden blocks. In a previous study, long-tailed macaques and olive baboons showed a superior performance when they were not rewarded with the same edible items they had to choose from (Schmitt and Fischer, 2011); this was interpreted as evidence that the monkeys may have problems with mentally representing the same edible items as two different things, i.e., as choice stimuli and as reward. Due to the at chance performance of the horses, we cannot draw any conclusions about how the horses may have mentally represented the choice stimuli.

Despite finding mainly null results, we believe that these results nevertheless may be of interest for researchers interested in comparative cognition. We refrain from claiming that our results provide sufficient evidence to assume a lack of quantity discrimination skills in horses. Previous studies demonstrated that horses discriminate between objects of different sizes (i.e., showing discrimination of continuous quantities; Hanggi, 2003) as well as discrete quantities such as 1 vs. 2 and 2 vs. 3 (Uller and Lewis, 2009). And indeed, some of our horses solved these as well as other quantity discriminations. However, we did not find a robust above chance performance on the group level. Yet, our problems to demonstrate quantity discriminations in the horses are in line with other reports of horses’ high susceptibility to changes in testing conditions (see Proops et al., 2009a and references therein). Whereas this alone is interesting (e.g., Agrillo and Petrazzini, 2012; MacLean et al., 2012), it would be preferable to identify the source of the different results. Unfortunately, we cannot identify a single cause, but in the following we will closely examine the differences between the study by Uller and Lewis (2009) and our study. Ultimately, we hope that this will be helpful for other researchers interested in quantity discriminations in horses (or other animals) to refine their methodological approaches and/or to develop new ideas and research questions.

In contrast to Uller and Lewis (2009), who tested a large number of horses at seven different locations throughout Essex, we tested a small group of horses only, housed at a single location in Germany. Because of this, each horse was tested only once in the previous study, whereas our subjects were tested repeatedly. It may be possible that Uller and Lewis’ horses were more motivated or that our horses may have become satiated over the course of the session. However, we did not find any evidence for performance decreases, neither within nor across sessions; yet, it is noteworthy that our horses took more time to choose on later trials within sessions (Yuki Henselek, pers. obs.).

In contrast to Uller and Lewis (2009), we tested Icelandic horses only. We are not aware of any studies reporting differences in the cognitive abilities of different breeds of horses, but mules (hybrids between horses and donkeys) performed better than ponies and donkeys in a learning task (Proops et al., 2009a); thus, there is a potential for cognitive differences between horse breeds. Noteworthy, dog breeds have been suggested to differ in cognitive abilities (e.g., Wobber et al., 2009; Jakovcevic et al., 2010). For instance, larger dogs seem to be more responsive to human pointing cues than smaller dogs (Helton and Helton, 2010), and various explanations have been raised to explain this difference: larger breeds may have been selected for working with humans and thus being more responsive to human pointing (Wobber et al., 2009), may possess a larger visual acuity or may simply have more experience with human gestures (Helton and Helton, 2010). Another difference was that Uller and Lewis conducted their tests in the subjects’ home stables, whereas we tested the horses in a test court inside a riding arena. Even though our setup may have been new to the subjects, the general surroundings were familiar for all horses and none of our subjects showed signs of distress. Horses in other studies using setups comparable to ours demonstrated their cognitive skills in a variety of tasks (e.g., Proops and McComb, 2010, 2012; Krüger et al., 2011), and we consider it unlikely that the general test surroundings can explain the different findings. Uller and Lewis offered entire apples that were put sequentially into buckets. We, in contrast, used apple slices only and positioned them simultaneously on two trays. Horses’ visual acuity is lower than humans’ (Timney and Keil, 1992), and consequently our presentation style may have been less conspicuous and demanded more attention to assess the number of items. Still, the apparent success of some subjects on some discriminations indicates that the horses could still see the difference. Furthermore, as one of our reviewers has pointed out, “apples may be natural objects categorized by horses in countable sets” and this may have facilitated the task for them. Unfortunately, whenever only a small number of horses are available and repeated trials per sessions are required, rewarding with entire apples is impossible because it could lead to rapid satiation. In future studies, an elegant solution to circumvent this problem could be to use artificial apples (such as those used by Uller and Lewis) as stimuli, but slices of apple as reward. Lastly, apples were baited out of sight in the Uller and Lewis study, whereas in our test the apples were visible during the entire trial. Speculatively, the hiding of the apples may have induced the horses to rely more on memory processes than purely perceptual processes, and this may have primed the horses’ attention toward the quantity of apples in each bucket.

Despite the possibility that some or all of the above mentioned factors may have contributed, we believe that two other points may be of particular importance: first, Uller and Lewis approached the horses while holding the buckets in their hands, whereas in our study, the horses had to approach the setup while the rewards rested at the ends of a 2.2 m long bench. Second, in our pre-test the horses learned to distinguish a tray with an apple from an empty tray, whereas others (including Schmitt and Fischer, 2011) had used pairs of stimuli with large quantity differences between them (e.g., 8 vs. 1). Theoretically, our horses may have learned to approach a tray with an apple rather than the tray with more apples, which may have disrupted their performance. For future studies it could be an alternative to conduct pre-training with a discrimination task in which subjects should discriminate between two quantities rather than training them to choose between one vs. zero. Zero has a special role in numerical competence and quantity discrimination tasks as it has the cardinal characteristic of “nothing” or “absence of items” which is more difficult to understand, even for chimpanzees (Biro and Matsuzawa, 2001).

Lastly, even though we and Uller and Lewis (2009) found at least tentative evidence for quantity discrimination abilities in a food-choice task, it may be worth discussing whether quantity discrimination tasks with food as stimuli are relevant for an animal that feeds on grass. A lack of ecological relevance may at least explain the lack of robustness of the animals’ performance. Equids are highly social animals (Linklater, 2000), whose social organization has been characterized by fission-fusion dynamics (Fischhoff et al., 2007). They recognize other individuals (Proops et al., 2009b) and dominance relationships have been shown to influence foraging decisions (Krüger and Flauger, 2008). It seems plausible that not only the identity, but also the number of herd members may influence foraging decisions of horses and future studies may take this into account. For instance, single female mosquitofish prefer to approach the larger of two shoals (Dadda et al., 2009), demonstrating the relevance of quantity and numerical discriminations in social processes.

In conclusion, using a foraging task we found only very limited evidence for quantity discrimination competences in horses, which is in contrast to a previous study (Uller and Lewis, 2009). Even though we do not doubt that horses are capable of differentiating between quantities, our study suggests that their competencies may be restricted to certain contexts and test conditions. This highlights the validity and importance of replication studies (Agrillo and Petrazzini, 2012; Perdue et al., 2012) to increase our understanding of the robustness of cognitive skills of non-human animals.

## Conflict of Interest Statement

The authors declare that the research was conducted in the absence of any commercial or financial relationships that could be construed as a potential conflict of interest.
